# The use and misuse of evolutionary psychology in online manosphere communities: The case of female mating strategies

**DOI:** 10.1017/ehs.2023.22

**Published:** 2023-08-30

**Authors:** Louis Bachaud, Sarah E. Johns

**Affiliations:** 1 School of Anthropology and Conservation, University of Kent, Canterbury, Kent, UK; 2 Laboratoire CECILLE, Université de Lille, Lille, France

**Keywords:** short-term mating, extra-pair mating, female mating strategies, feminism, manosphere, incels

## Abstract

While early evolutionary accounts of female sexuality insisted on coyness and monogamous tendencies, evidence from the field of primatology started challenging those assumptions in the 1970s. Decades later, there exist many competing and overlapping hypotheses stressing the potential fitness benefits of female short-term and extra-pair mating. Female mammals are now seen as enacting varied and flexible reproductive strategies. This is both a victory for science, with a better fit between theory and reality, and for feminism, with the downfall of narrow stereotypes about female sexuality. However, evolutionary hypotheses on female mating strategies are routinely invoked among the antifeminist online communities collectively known as ‘the manosphere’. Based on extensive qualitative analysis of manosphere discourse, this study shows how these hypotheses are sometimes interpreted in misogynistic online spaces. Indeed, evolutionary scholars might be surprised to see sexist worldviews reinforced by the ‘dual mating strategy’ and ‘sexy son’ hypotheses, or by the latest research on the ovulatory cycle. The manosphere has its own version of evolutionary psychology, mingling cutting-edge scientific theories and hypotheses with personal narratives, sexual double standards and misogynistic beliefs. After analysing this phenomenon, this article suggests ways to mitigate it.

## Graphical abstract

**Figure:**
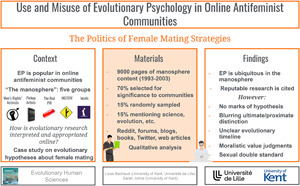

**Social media summary:** Evolutionary psychology is popular in the manosphere. This study examines Internet uses of evolutionary hypotheses.

## 1. Introduction

Since their inception, evolutionary approaches to human behaviour have been appropriated for a variety of ideological purposes (Alexander & Numbers, 2010). For example, nineteenth-century feminists used Darwin's account of sexual selection through female choice to make the case for women's autonomy (Hamlin, 2014). In the meantime, Darwinian/Spencerian ‘survival of the fittest’ was invoked by conservatives to justify *laissez-faire* in economic and social policy (Hofstadter, 1944). More recently, evolutionary hypotheses, such as those focused on female mating strategies, have been invoked in antifeminist online spaces (collectively known as ‘the manosphere’) to support misogynistic worldviews. These (mis)understandings of evolutionary psychology (EP) should be extremely concerning to those working in the field because legitimate scientific hypotheses are routinely used to justify disdain towards women. Here, we present the results of a qualitative study of how evolutionary science is used and misused by in the manosphere, and present some suggestions to mitigate this issue.

All definitions of the manosphere agree on three points: (1) the manosphere is a constellation of loosely related groups and movements; (2) it is principally Internet-based; and (3) those groups are united by a masculine perspective and the defence of male interests. While it started as an Internet neologism, ‘manosphere’ now has an entry in the Cambridge Dictionary: ‘websites and internet discussion groups that are concerned with men's interests and rights as opposed to women's, often connected with opposition to feminism or dislike of women’ (Cambridge Dictionary, 2023).

Based on long-term observation of the manosphere, we follow a typology composed of five main communities: Men's Rights’ Activists (MRAs), Pickup-Artists (PUAs), Men Going Their Own Way (MGTOW), the Red Pill (TRP) and incels (‘involuntary celibates’) – see Table 1 for each group's diagnosis of gender relations in society and their reaction to it. This is a popular typology in recent research (Ribeiro et al., 2021; Krendel et al., 2022; Rothermel et al., 2022).

**Table 1.** Overview of the five main manosphere communities and their associated beliefs

Group	Diagnosis	Reactions
Men's rights’ activists (MRAs)	Feminism has established a societal and legal system that is stacked against men, in which men's problems are ignored or downplayed	Recruitment, indignation mobilisation, activism and advocating for policy change
Pickup artists (PUAs)	Approach gender relations with transactional logic where seduction relies on the (economic) value of a PUA, which is based on his performance of masculinity	Self-improvement and manipulation of women as a way for men to seduce women [PUAs call their seduction techniques ‘Game’]
Men Going Their Own Way (MGTOW)	See women (enabled by feminism) as manipulative and dangerous to men's autonomy, including financial autonomy, and society as gynocentric (overly focused on women)	Idealised withdrawal from society and self-reliance, limiting relations with women, especially legally binding ones, and avoiding (all) interactions with women altogether
The Red Pill (TRP)	Economise relationships and believe in a sexual marketplace, in which everyone has a certain sexual market value. See feminism as the ‘sexual strategy’ of women to gain higher value males/mates and perceive the ‘sexual marketplace’ as stacked against them as a result	Manipulation and ‘Game’ to contend in the sexual marketplace, ‘the Red Pill’ as men's sexual strategy, and an ‘awakening’ to a previously hidden truth
Misogynist [‘blackpilled’] incels	Believe their looks and feminism to be the reason they are rejected by women. Consider rejection as unjust victimisation of their identity and that some part of their humanity is unfulfilled, rendering them ‘subhuman’ [incels call this set of beliefs ‘the blackpill’]	Nihilism that can result in a variety of violent or abusive reactions (e.g. poverty sex-tourism, self-harm, societal insurrection, sexual violence, or mass violence), each asserting dominance over and punishing women.

*Source:* replicated from Rothermel et al. (2022: 133–135; bracket comment ours).

As shown in Table 1, discussions of (heterosexual) sex and relationships feature prominently in the manosphere. Given its strong focus on sex research, EP enjoys widespread popularity among those online communities. Paradoxically, research on female sexuality is especially prevalent in these male spaces. Indeed, online discussions on sexual selection by female choice, evolved mate preferences, or female mating strategies abound.

To understand the phenomenon, this study focuses on the online interpretations and uses of evolutionary research surrounding female mating strategies, firstly because this is a popular topic in the manosphere, and secondly because much of this research is usually perceived in scientific history as an important feminist achievement – rooting out male bias from evolutionary science.

By the 1970s, contradicting Darwin's famous description of female animals as ‘coy’ and ‘less eager to mate than the male’ (Darwin, 1871: 273), observations showed that females in primate species, such as macaques and bonobos, did not appear to be very coy nor very selective (Hrdy, 1981). Moreover, as more evidence revealed the prevalence of extra-pair mating in supposedly monogamous species, particularly in birds (Birkhead et al., 1987), the same question was raised: what's in it for females’ fitness (Kempenaers et al., 1997)? Nowadays, competing and overlapping hypotheses are refined and empirically tested in the field of EP, to determine how the propensity for casual or extradyadic sex evolved in women, and identify the relevant cues and mechanisms underlying those behaviours (for a review of hypotheses, see Greiling & Buss, 2000).

Overall, this reappraisal of female sexuality can be hailed as a major advance for the biological sciences, bridging the gap between theory and empirical observations. However, it is also important for feminism, with the erosion of stereotypical views on female sexuality (Cooke, 2022). Women (and other female mammals) are not uniformly coy and passive. They are in fact implementing varied and flexible mating strategies and can, on occasion, be as sexually assertive as males. Given this legacy, evolutionary scholars might be quite surprised to see evolutionary research on female mating strategies appropriated in misogynistic ways.

Before proceeding with the analysis, it should be noted that the manosphere is a broad constellation of groups and ideas, with members from all over the world. Furthermore, not all communities are equally keen on EP. It is for example less popular among Men's Rights’ Activists, who tend to favour sociocultural analyses. Even inside communities, positions vary greatly. Examples from all groups are provided, yet the aim of this paper is not to dwell on the many ideological nuances and divergences between manosphere communities, but to draw the attention of the EP community towards their common use of evolutionary science.

## 2. Methods

In order to analyse the role of evolutionary science in the manosphere, an extensive qualitative study of manosphere discourse was conducted. A selection of discourse, or ‘corpus’, was constituted, spanning three decades (1993–2022), and evenly divided among the five communities. Most of the material was selected for its importance to manosphere communities (‘Quality Content’ section – 70%). This consisted of ‘top posts’, ‘most popular threads’, most viewed videos, etc. Each addition was justified if the website, platform, or book was central to the community, i.e. consistently cited on other manosphere websites, and/or identified as such by researchers. Another section consisted of material selected for its use of evolutionary science and relevance to the analysis (‘Other Related Material’ section – 15%). A third section was randomly sampled on Reddit and forums for representativity (‘Random Sample’ section – 15%). The random sampling schedule and procedures are detailed in Supplementary Material S2. The whole content selection process is summarised in Supplementary Material S3. After transcription of audio and video content, the material covers 9000 pages and contains Reddit posts, forum threads, e-books, books, blogs posts, web articles, online encyclopedia entries, tweets and YouTube videos. The complete list of corpus material is provided in Table S1 (Supplementary Material).

The corpus analysis was guided by several research questions. Those research questions pertained to evolutionary science and its role in manosphere ideology and discourse. What academic concepts and ideas get appropriated online? To what end? Is the scientific literature thus distorted or misinterpreted? In what ways? To answer those questions, information from each corpus document was extracted, classified, and summarised in a standardised template, as well as being labelled with different ‘tags’ – a form of thematic qualitative coding. An example of filled template is reproduced in Supplementary Material S4.

Through the example of online discussions of female mating strategies, this article presents some of the qualitative analysis findings on the role of EP in the manosphere. This is a targeted case study, drawn from the broader findings of the extensive corpus analysis.

To protect personal identities when quoting, Internet users are anonymised, and no hyperlinks to their comments are provided. Their quotes will only be referenced with their manosphere community and type of platform, e.g. (Incel, forum). On the other hand, content from public manosphere figures, such as bloggers and writers, is cited normally. The quoted material contains offensive and hateful language; however, since this article aims at raising awareness, we believe that it is important to depict these online communities through their own words. These data protection and dissemination measures were approved by the Universities of Kent and Lille's review boards (approval numbers respectively 8-PGR-20/21 and QSMDC 2021-478-S91).

## 3. Results

### Evolutionary psychology in the manosphere

Evolutionary psychology is ubiquitous in the manosphere. This has been mentioned by social scientists (Ging, 2019; Van Valkenburgh, 2018), but has not been the subject of much analysis by evolutionary scholars themselves. The first of this kind was an article reviewing Pickup-Artist seduction guides and concluding ‘that many of these claims are in fact grounded in solid empirical findings from social, physiological, and evolutionary psychology.’ (Oesch & Miklousic, 2012). A recent study also briefly commented on misuses of EP by incels (Baselice, 2023: 12–13).

Furthermore, a recent overview of manosphere research included EP as a dominant theme in manosphere discourse:

These formations are united in their antipathy toward feminism, their reliance on evolutionary psychology and their belief that Western civilisation is under threat. (Ging & Murphy, 2021: 1)

Indeed, the founding manifesto of the Red Pill community recognises its reliance on EP:

A large portion of Red Pill discussion revolves around evolutionary psychology. Understanding the facets of this psychology are key to developing a good sexual strategy. Because this strategy is useful not only in gaining the attention of the opposite sex, but continuing relationships, having children, and maximizing your own happiness throughout life. (The Red Pill, Reddit).

In the Pickup-Artist community as well, several seduction guides are grounded in Darwinian principles. For example, *The Mystery Method* opens with a gloomy Darwinian injunction, ‘Nature will unapologetically weed your genes out of existence if you don't take action and learn how to attract women now’ (Mistery, 2005: viii), while Nick Savoy's *Magic Bullets* includes ‘A quick primer on evolutionary biology’ (2007: 25).

No manosphere community shares and discusses more EP research and concepts than the incel community, in particular those who adhere to the set of beliefs called ‘the blackpill’ (for a definition, see Table 1, and Radicalisation Awareness Network, 2021). On Incels.wiki, the online incel encyclopedia, the ties between the blackpill and EP are made clear:

The Scientific Blackpill is about understanding the nature of human social and sexual behavior with a particular focus on evolutionary psychological perspectives. (‘Scientific Blackpill’, n.d.)

The entry then goes on to cite and summarise more than 100 scientific studies, from mainstream journals in the evolutionary and behavioural sciences.

Countless similar examples could be provided. As a case study, this article will now be focusing on the depiction of female mating strategies in manosphere communities, but their use of EP is indeed a ubiquitous phenomenon.

### The dual mating strategy hypothesis

In the manosphere, the most popular hypothesis to explain female extra-pair mating is the dual mating strategy hypothesis. According to this hypothesis, extra-pair mating could have been adaptive for ancestral women if they managed to secure investment from a regular partner, while mating with affair partners would provide ‘fitter’ genes for their offspring (Weisberg & Kim, 2021). Most of the empirical research derived from the hypothesis investigates the ovulatory cycle and whether female mate preference shifts around ovulation. For example, do women fantasise about, or pursue more attractive men at that time? (For an evidentiary summary, see Gildersleeve et al., 2014; Gangestad & Haselton, 2015.)

Like other research on female sexuality, the dual mating strategy hypothesis is hailed in the manosphere as proof that women's supposed fidelity and desire for monogamy is a myth:

You pretty much destroy the fairy tale that men are the polygamous ones and women merely the hypergamous ones. As IF it is ONLY those those bad men who cheat due to being sexual beasts. Not Miss Snowflake. […] to get rid of the fantasy that women don't have dual mating strategies is part of main path to freedom PERIOD! (MGTOW, forum)

In fact, this hypothesis is sometimes described as a revelation for men:

It's 2019, we all know the secret females have been hiding for over a million years now. DUAL MATING STRATEGY. Fuck the alphas [alpha males], suck resources and attention from all others. (MGTOW, Reddit)

Manosphere renditions of the dual mating strategy hypothesis reveal how evolutionary hypotheses are understood and shared online.

There is an observed dualistic mating strategy observed in primates and anecdotally in humans. Women have two motives for using sex. Primal: In an intimate reproductive urge to obtain genes from a partner. Passion and horniness. Transactional: in a survivalist exchange to obtain resources from a partner. Female Bonobos will trade sex for food, and women will marry rich men they are not sexually attracted to. (The Red Pill, Reddit)

There is no mark of hypothesis, in fact, the dual mating strategy *hypothesis* is not described as such in the manosphere. Here, it is just described as a scientific and ‘observed’ strategy that females engage in. This other post from Reddit is even more peremptory:

If you aren't new here, then you would know about women's dual mating strategy – long term dating strategy and short term dating strategy. Before someone objects that women do not have a dual mating strategy, I would like to state that this is in fact false. Women do have a dual mating strategy as evidenced by [Durkee et al., 2019] (https://journals.sagepub.com/doi/full/10.1177/1474704919852918). (The Red Pill, Reddit).

Not only does this poster profess the undoubted truth of the hypothesis, but he also assumes that everyone in the community will be familiar with it, a testimony to the popularity of EP within these groups. And yet the study cited by the poster had mixed results, and does not even mention the dual mating strategy hypothesis. A final example from Reddit illustrates this peremptoriness, with a monolithic view of female behaviour, which is held to be constant ‘in the wild’ and in contemporary societies:

Lots of good solid research has been done on mating preferences and the menstrual cycle in women. So much that I'm not sure where to begin …

In the wild females will seek out two basic types of mates: one's for the long term, and one's that can deliver the best genetics. […] If your woman is every going to cheat, it's most likely going to be during ovulation. In the wild females will bond with a mate who they see as being a good caregiver for their children, and secretly sneak off during ovulation to get pregnant with with the one they believe carries good genetics. (Pickup-Artist, Reddit)

In the example above, women seem to be consciously selecting mates whom ‘they believe carries good genetics’. This is obviously false, and is a classic case of mistaking the ultimate (mate with ‘good genetics’) for the proximate (attractive mate). However, this is a mistake that some in the manosphere try to avoid, for example Red Pill writer and pundit Rollo Tomassi,

I want to stress again that (most) women do not have some consciously constructed and recognized master plan to enact this cycle and deliberately trap men into it. Rather, the motivations for this behavior and the accompanying social rationales invented to justify it are an unconscious process. For the most part, women are unaware of this dynamic, but are nonetheless subject to its influence. ***For a female of any species to facilitate a methodology for breeding with the best genetic partner she's able to attract AND to ensure her own and her offspring's survival with the best provisioning partner; this is an evolutionary jackpot***. (Tomassi, 2011)

This level of precaution is quite rare, and it is unclear how widespread it is among ordinary members of manosphere communities. In fact, taking the ‘gene's eye view’ – writing as if people consciously act to maximise their fitness – is also commonplace in EP literature, e.g.

The current leading hypothesis for why women have affairs posits that women have adaptations for securing investment from one man while cuckolding him in order to obtain good genes from an affair partner. (Buss et al., 2017: 147)

When formulated this way, it looks as if women actively cheat ‘in order to obtain good genes’. Here, these ‘gene's eye view’ redactional shortcuts end up exactly mirroring common manosphere beliefs. Indeed, there is an adage in the manosphere called ‘Alpha Fux, Beta Bux’, abbreviated as AF/BB. This acronym designates the supposed tendency for women to stay in relationships with unattractive but stable and caring men who provide for them (‘betas’), while cheating on them with more attractive men (‘alphas’). Hence the reaction of this incel after discovering the dual mating strategy hypothesis,

Doesn't this read like some blackpill straight out of an incel forum? jfl [just fucking LOL] it's exactly alpha fucks, beta bucks. (Incel, forum)

People do not consciously act in their genes’ best interests. Yet, the use of the term ‘strategy’ in the evolutionary literature misleadingly reinforces that impression. Indeed, it can refer to two different levels of strategies. On the one hand, strategies employed by individuals to achieve some (proximate) goal, and on the other hand evolutionary strategies competing against others in the (ultimate) arena of natural and sexual selection. This conflation might not be problematic in most species; when behavioural ecologists discuss strategies in fish or insects, these are clearly evolutionary-level strategies. But in species with higher cognitive faculties, and the capacity of conscious strategising, this creates confusion. Thus, in the Red Pill community's founding manifesto, feminism is described as a way for women to enable their own sexual strategies,

**Feminism is a sexual strategy.** It puts women into the best position they can find, to select mates, to determine when they want to switch mates, to locate the best dna possible, and to garner the most resources they can individually achieve. (The Red Pill, Reddit).

This is inspired by EP terminology and concepts – ‘sexual strategy’, ‘switch mates’, ‘best dna’ – and confuses (proximate) politics with (ultimate) fitness, through the use of the ambiguous term ‘strategy’. In fact, in order to mirror feminism's supposed sexual agenda, the Red Pill community purports to provide ‘a sexual strategy in a culture increasingly lacking a positive identity for men’ (The Red Pill, Reddit). Hence its reliance on EP, because ‘understanding the facets of this psychology are key to developing a good sexual strategy’ (The Red Pill, Reddit).

Certainly, Darwinian theory pits antagonistic evolutionary interests against each other, with sibling rivalry and parent–offspring conflict for example (Trivers, 1974). And the evolution of sex has also been shaped by the diverging interests of males and females (Arnqvist & Lowe, 2005). Yet, these are conflicts between ultimate fitness interests – ‘strategies’ as conceived in game theory – not between conscious strategies. In the manosphere, this fact is often lost. There, through the use of EP terminology, an antagonistic Darwinian framework is mapped onto contemporary gender politics, depicting feminism and antifeminism as female and male reproductive ‘strategies’ pitted against each other.

Our analysis reveals the prevalence of EP hypotheses on female mating in the manosphere, often drawn from reputable scientific sources. However, their manosphere renditions mostly obscure the fact that female mating strategies are hypothetical, unconscious, and supposed to have evolved aggregately over time. Interestingly, hypotheses on the evolution of male sexuality are not discussed much in the manosphere: as if only women were strategising fitness-maximising animals. Moreover, hypotheses on female sexuality are received in an emotional, moralistic, and conservative framework, causing biased interpretations of the scientific literature.

### Negative attitudes towards female sexuality

Although extra-pair mating is just a matter of scientific inquiry for evolutionary scholars, that is not the case in the manosphere, where emotions on the topic run high. As a space where men can share their dating, relationship, and marriage experience, the manosphere is home to a lot of pain, anger, and bitterness. Apart from conflictual divorces, one of the most frequent source of resentment is being cheated on by a female partner. In fact, in a MGTOW discussion entitled ‘What are the worst lies a woman ever told you?’, one finds dozens of testimonies of men who were cheated on by wives or girlfriends. To some men reading those testimonies, realising that their misfortunes were shared by others has a comforting effect:

I cant tell you how therapeutic it is to my conscience to hear others listing the same lies I've heard day in and day out for years and years from multiple women. (MGTOW, forum)

For others, it just paints a truer picture of reality, showing that women are no better than men when it comes to honesty and fidelity:

Men have been taught to believe that females are true and kind and just and that it is our inability as men to restrain our base urges to dishonesty, violence and fucking around that destroy marriages when, in fact, it is just as often the females who lie, cheat and lash out against us. (MGTOW, forum)

Yet, for others, the fine line between anecdotes and generalisation is easily crossed:

She turned out to be a very selfish, self-centered, arrogant, cheating bitch but then again, aren't they all like that? (MGTOW, forum)

The following excerpt from a Red Pill redditor perfectly illustrates that dynamic: I got cheated on, *ergo*, all women are cheaters:

I was in a LTR [Long-Term Relationship] and applying to med school. […] Fast forward to a month after I find out I didn't get in anywhere, and all of a sudden she's tearfully confessing to cheating six month ago and telling me how we shouldn't be together because she doesn't know if she loves me anymore.

Long story short: AWALT [All Women Are Like That]. No exceptions. (The Red Pill, Reddit)

Evolutionary thinking thus becomes an easy way to support generalisations about women. Men's Rights lawyer Roy Den Hollander married a Russian woman who turned out to be a sex worker, an experience that scarred him for life. He even created a website to share his 1500-page account of the story (https://web.archive.org/web/20230130132220/http://been-scammed.com/main/). There, one finds an evolutionary explanation for female extra-pair mating. In typical manosphere fashion, his narrative is not presented as speculative, nor is it substantiated by empirical data,

A woman's drive for sex and economic support, which is the modern-day form of protection, made infidelity a way of life for her. Females spread their bets, so if one man bites the dust, either physically or economically, she still had other beaus to depend on. To keep her beaus tied to her, she needed to cheat on all of them but still convince each one with her tears, entreaties and sex that he was the only one. Over millions of years, natural selection eliminated the faithful females, since they tended to die out with only one male protecting and supporting them. That left modern-day man with only a huge pool of hos – billions of them. (Den Hollander, n.d.: 19–20)

This exemplifies another key difference between the scientific literature and the manosphere: moral judgments rarely appear in the evolutionary literature. Nature being fundamentally amoral, there are no normative or condemnatory overtones in the study of extra-pair mating. This scientific neutrality is in stark contrast with Den Hollander's sexism. Here, as elsewhere in the manosphere, the author's use of evolutionary reasoning is suffused with personal grief and extreme misogyny.

More generally, attitudes towards female sexual agency in the manosphere tend to be very negative. The sexual liberation spurred by feminism is routinely bemoaned by the more traditionalist factions as the downfall of traditional (and chaste) femininity. When primatologists wrote about females’ being potentially as competitive or sexually assertive as males, this was seen as a much-needed correction of scientific bias, and as a feminist breakthrough. Yet, if one sees female sexual freedom as undesirable, then the interpretation is the complete opposite, as is often the case in the manosphere:

This study proves why the sexual revolution was by far one the biggest mistakes the west could've made. Paired with biological drives to cheat, and because you cannot run away from your genetically hard-wired desires, giving women complete sexual freedom was opening pandora's box. (Incel, forum)

Thus, depending on values, the same evolutionary hypothesis can be interpreted in completely opposite ways. For example, incels regard themselves as unattractive genetic misfits, and as the victims of a contemporary society where female sexuality is unhinged, making for a specific interpretation of evolutionary hypotheses. Their example demonstrates further how emotions and ideology fuel sexist interpretations of evolutionary scholarship.

One discussion thread is particularly revealing. The originator of the thread is citing abundantly from the Wikipedia.org entry on concealed ovulation. For each section, he sums up the encyclopedia article for his fellow forum members. Thus, this Wikipedia passage:

Schröder in his review writes that Benshoof and Thornhill hypothesized that estrus became hidden after monogamous relationships became the norm in *Homo erectus*. Concealed ovulation allowed the woman to mate secretly at times with a genetically superior man, and thus gain the ‘benefit of his genes for her offspring, while still retaining the benefits of the pair bond with her usual sexual partner. (‘Concealed ovulation’, n.d.)…

becomes,

Concealed ovulation allows women to breed with genetically superior men (aka chads). (Incel, forum)

Gone are all the marks of hypothesis (‘hypothesized’, ‘would’), while the tense of the sentence has also changed: speculations about the past become a generalisation in the present. As for another evolutionary hypothesis about meat being exchanged for sex after hunting (see Schröder, 1993: 383–384), he sums it up somewhat anachronistically,

Concealed ovulation was partly born out of prostitution. (Incel, forum)

The incel poster concludes by stating:

It's absolutely undeniable that women have evolutionary adaptations geared towards cheating, which also shows how prevalent cheating was throughout history (and still is). (Incel, forum)

In his summation, he leaps from mere hypotheses to ‘absolute undeniability’ – no small leap. As elsewhere, the scientific clout of EP is invoked as a sign of undoubted truth. The reactions of the incels who read his post reveal how common forum users interpret evolutionary hypotheses.

nature is cruel tbh [to be honest]. (Incel, forum)

This user is just acknowledging a basic fact of Darwinism: evolutionary processes can select for behaviour deemed immoral as well as moral. It is one of the rare occurrences of a non-sexist reaction.

Women are absolute scum. (Incel, forum)

The amorality of nature is not recognised by all though.

This is a hard pill to swallow. Even if you ascend [lose your virginity], she will still look for Chad. (Incel, forum)

This comment is voicing a common fear among incels. Even if one manages to find a girlfriend someday, she will be unfaithful – after all, isn't this in her best genetic interest? Since incels perceive themselves as men of low genetic quality, they feel especially concerned by the dual mating strategy hypothesis, which posits that female extra-pair mating might have evolved through mating with ‘higher-quality’ mates. Yet, rather than reject a hypothesis which would look unpalatable to them, they embrace it. Indeed, incel or ‘blackpill’ ideology is based on the idea that incels’ destinies are out of their hands, determined instead by powerful social, genetic, and evolutionary forces (Brzuszkiewicz, 2020: 12–13). Chief among those forces is female mate selection, which to them is synonymous with female rejection.

Hence, their fascination for hypotheses on the evolution of female sexuality such as the dual-mating hypothesis, or for the ‘good genes’ and ‘sexy son’ hypotheses models of sexual selection (for meta-analysis on these two hypotheses, see Prokop et al., 2012). Evolutionary scholars might be surprised to learn that the erudite debate between Fisherian and ‘good genes’ models of sexual selection is also echoed in the ‘incelosphere’, on the Incel Wiki (‘Good genes hypotheses’, n.d.) By stressing the evolutionary importance for females of selecting healthy and attractive mates, these hypotheses vindicate incel worldview, and do so with the authority of peer-reviewed science. They are thus shared and commented with glee:

I find it hilarious that a wikipedia article is saying things that sound like posts from a blackpilled forum. (Incel, forum)

Nice to have scientific peer reviewed sources for foids [women] being cum whores by nature. (Incel, forum)

Yet, they are also received with despair. Indeed, as men whose sexual and romantic aspirations are unmet, incels have a very particular relationship towards sex research. They are avid consumers of it, and use it to explain their celibacy. But it also reinforces their fatalism, and their view that genetics and biology entirely determine dating and relationship success. They often refer to data and research about sex as ‘suicide fuel’ or ‘suifuel’ (suicide is not uncommon in the community, see Daly & Laskovtsov, 2021). Thus, after reading a post about sperm competition and the dual mating strategy hypothesis, a user writes,

I knew those two facts but now that i associate them **it just destroyed me mentally.** (Incel, forum)

The line between despair and hatred is a thin one, as shown by this incel's reaction to the same post,

One of the most brutal suifuels of all times. I'll just link this post anytime someone asks me why we should hate women. (Incel, forum)

Incels resent the fact that other people have sex. This bitterness brings some of them towards the extremes of suicide and/or of harming others (for an overview of incel-related attacks, see Hoffman et al., 2020). In that context, any type of sex research is bound to infuriate them. But that is even more true of research on the evolution of concealed ovulation, female orgasms or short-term and extra-pair mating. Since these insist on the prominent evolutionary role of females' agency and multiple mating, incels see them as cruel reminders of their rejection by women and of their own sexlessness. Thus, it is certainly among incels, who are the most avid consumers of EP in the manosphere, that reactions to such research are the most starkly misogynistic:

So women are hardwired to be cheating whores there is no escape. (Incel, forum)

The punishment for adultery in Islam is death. […] I realize now that this is a good and necessary thing to prevent degeneracy and cuckolding which is in the genetic code of femoids [women]. (Incel, forum)

High IQ thread. Further proves that foids [women] are brainless primates that can't control their biological behaviour that could lead to other people suffering from their actions. This is why they shouldn't be allowed to control society or else this world will go to shit. (Incel, forum)

Before concluding the analysis, one bias must be pointed out here: there is no mention of male instincts and male behaviour. If women's propension for extra-pair mating makes them ‘brainless primates’, what about the well-established *male* propension? Are men ‘absolute scum’ as well for being equipped with mechanisms coming from an evolutionary legacy of short-term and extra-pair mating? This is, to our knowledge, not a line of reasoning ever found in the manosphere. Thus, the age-old sexual double standard is reproduced in these seemingly scientific discussions: regarding men, inclination towards short-term and extra-pair mating is so evident and unproblematic that it does not warrant any comments or explanation, while in women it is a major concern, warranting competing hypotheses, and prompting scathing condemnations. Evolutionary explanations are thus used to depict a monolithic and inflexible view of female ‘biological behaviour’, but are rarely applied to male behaviour.

## 4. Discussion

It would be comforting to dismiss the sexist uses of EP as stemming from outdated scholarship or from crude pseudoscience. Yet, it is clear that manosphere communities often draw on reputable research from peer-reviewed journals, making the picture much more complicated. Beyond the illustrative examples cited in this paper, the detailed analysis of discussions on female mating strategies has yielded several key findings.

Firstly, the manosphere is undeniably very fond of evolutionary theories about the evolution of female sexuality. The Incels.wiki entries on ‘strategic pluralism’, ‘good genes’ and ‘Fisherian runaway’ (n.d.) show the attention devoted by some to competing evolutionary hypotheses and their latest articulations. In fact, those theories seem to perfectly espouse some manosphere beliefs such as ‘Alpha Fux/Beta Bux’, or the whole incel ‘blackpill’ worldview, so much so that some consider EP and ideology as two sides of the same coin:

The blackpill, at its core, is just basic evolutionary science. the people denying it are no different from creationists. (Incel, Reddit)

However, as our analysis revealed, a lot of subtle and not-so-subtle shifts occur between EP and its manosphere version. Mostly gone are the marks of hypothesising. So are the precautions about using the ‘gene's eye view’ shortcut, or about the conditional nature of instincts. The timeline also changes: while academic hypotheses dwell on the aggregate behaviour of our ancestors over millennia, their manosphere versions are more unclear on that aspect.

This is coupled with a total absence of discussion on male sexuality and its evolutionary underpinnings; an omission which creates a totally different and biased account of reality. To say the least, the vision of male nature presented by EP is not very flattering compared with its female counterpart. Yet, paradoxically, by omitting all those unsavoury aspects and focusing exclusively on female behaviour, EP is still routinely employed by manosphere groups to support their worldview and skewed vision of sex differences.

The most striking difference between academic writing and the manosphere remains the presence of moral judgments in the manosphere. While scientists try to explain and understand, manosphere activists often bemoan, condemn, and vituperate. Sexist interpretations abound, framed by emotions and ideology. We assume that evolutionary psychologists would be looking for ways to mitigate the hate and misogyny found in some of these interpretations.

### Recommendations

If these communities are already prejudiced against women, and hold moralistic views on female sexuality, can evolutionary sex researchers really avoid seeing their work being misinterpreted? Probably not. However, they can take steps to make such interpretations more difficult, and to ensure their own language does not unnecessarily reflect that of the manosphere.

For example, the use of the verb ‘cuckold’ in reputable academic writing is unfortunate. Firstly, because this is a sexist term. Indeed, this opprobrious label only concerns men whose wives are unfaithful, but there is no equivalent for the wife of a philanderer – a clear case of sexual double standard, a point already made by biologist Anne Fausto-Sterling (Fausto-Sterling, 1985). Secondly, the term ‘cuckold’ has taken new importance online. Its abbreviation ‘cuck’ has become a popular epithet used to derogate one's opponents. In manosphere and Alt-Right parlance, ‘cucks’ are weak men, those who support liberal causes and feminism, or more generally anyone on the left (Kosse, 2022). Therefore, the standards of scientific writing should dictate the abandonment of a term which has traditionally been gender-biased and morally loaded, and is now increasingly politically charged. On a similar note, the use of the phrase ‘genetically superior men’ in the Wikipedia.org page on concealed ovulation espouses incel beliefs so perfectly that it is no wonder the incel poster wrote: ‘I can't believe this article is even up on such a mainstream platform’ (Incel, forum).

Finally, maybe a morally loaded term such as ‘infidelity’ could be avoided – as was the case in the present article. Obviously, it is impossible for the EP literature to use only neutral terms. Indeed, it studies all areas of human behaviour, all of which, like violence, cheating, or solidarity, come with their own moral connotations. However, given the lingering prevalence of sexual double-standards and the intensity of moral attitudes towards sex – two things that EP itself can very well shed light on (Zaikman & Marks, 2017; Farvid, 2021) – maybe sex research should be mindful to adopt particularly neutral language. In a similar logic, we avoided using the term ‘promiscuity’ in this article.

Furthermore, although metaphors and ‘gene's eye view’ simplifications are useful tools to translate evolutionary reasoning, they should always be accompanied with reminders that (1) people do not act consciously in their genes’ interests, i.e. restating the ultimate/proximate distinction, and (2) hypotheses dwell on *past* behaviour that could have selected for adaptations still present in contemporary humans, which does *not* imply that such behaviour is still occurring today (for example, many hypotheses pertain to hunting, when most humans do not rely on hunting for food anymore). While these reminders are commonplace in textbooks and popular EP books, they are often lacking in the more specialised research papers. Presumably, scholars do not feel the need to restate such obvious things to their esteemed colleagues. What our analysis reveals however, is that these articles are also routinely read, shared and discussed by online communities. Moreover, in abstracts, titles and conclusions, academic publishing also encourages the communication of results in very definite terms. This contributes to simplistic understandings of empirical findings, such as monocausal explanations for complex phenomena, or ignorance of effect sizes.

Beyond these suggested changes to academic writing, EP scholars might decide to engage directly with the issue, calling out or debunking biased interpretations of their research.

This article is just the beginning, as there are many areas of evolutionary science that garner substantial attention from the manosphere. For example, research on mate preferences or on the behavioural effects of hormones. An article debunking the claims of online body language ‘experts’ was recently published in this journal (Denault & Zloteanu, 2022).

Ultimately, this might not contribute to mitigating the prevalence of EP in manosphere communities – after all, EP is a rich and blossoming discipline. However, it would at least make it harder for serious scholarship to get assimilated by the general public to reactionary and misogynistic discourse.

### Limitations and future research

This research is based on analysis of mainly online content, which comes with inherent limitations. For example, little is certain about the pseudonymous Internet users of the manosphere, apart from their being mostly English-speaking men. Having more sociodemographic information would shed light on their understanding of evolutionary science – especially age, level of education, scientific education and religious belief, which are all predictors of scientific literacy and/or acceptance of evolution (Miller, 2010; Hawley et al., 2011; Miller et al., 2022).

Such qualitative analyses naturally gravitate towards the most active and vocal members of a given community. Indeed, a quantitative study on a manosphere forum found that half the posts were made by less than 1% of registered users (Wright et al., 2020). Similarly, radical communities receive more scrutiny than moderate ones. This is especially true in manosphere studies, where research on incels bloomed following the rise in incel-inspired mass killings (going from one study in 2015 to 32 in 2020, see Prøitz et al., 2022). Even if this article focused on misogynist or ‘blackpilled’ incels as a case study, this does not imply that all manosphere-affiliated groups and people agree with the extreme views depicted. Moreover, some of these online communities produce deliberately provocative or inflammatory comments (i.e. ‘trolling’ or ‘shitposting’), which further complicates analyses. From online discourse alone, it is thus particularly difficult to determine the prevalence and earnestness of certain beliefs. Additional research will establish a more detailed and nuanced account of the phenomenon. To obtain this more comprehensive picture, we have designed a large-scale manosphere survey, gathering responses from a broad range of manosphere users, and including relevant sociodemographic variables. It aims at assessing the level of evolutionary scientific literacy in the manosphere, and will further the present analysis.

## 5. Conclusion

Online manosphere groups routinely invoke, share and discuss cutting-edge evolutionary research. The clout of EP is used as a token of authority, as the conjectural nature of hypotheses is often obscured. An antagonistic Darwinian framework is sometimes imported from evolutionary theory to gender politics, conflating ultimate reproductive strategies and proximate behaviour. The sexual double standard is pervasive and underlies negative interpretations of research stressing female sexual agency and assertiveness. Our analysis reveals how easily sex research is interpreted through judgmental and misogynistic lenses. There are two avenues for concerned scholars to combat that. The first one is upstream, by insisting on neutral and careful scientific writing. The second is downstream, by engaging with popular uses and interpretations of evolutionary research, through debunking and analysis for example. We hope this paper will raise awareness, and spur debates and advances along those lines in the EP community.

## Data Availability

The corpus material is considered to be ‘personal data’ under GDPR regulations. In order to retrieve, store, and cite it, a GDPR derogation had to be filed on our behalf by the University of Lille's Data Protection Officer under the provisions of GDPR's article 89 (submission no. DPO 2021-34). According to the law's principle, the material is only to be stored for the duration of the study, and cannot be shared with third parties.

## Supplementary material

For supplementary material accompanying this paper visit https://doi.org/10.1017/ehs.2023.22. click here to view supplementary material
