# Supplementary material for: The use and misuse of evolutionary psychology in online manosphere communities: The case of female mating strategies
Source: Evol Hum Sci. 2023 Aug 30;5:e28. doi: 10.1017/ehs.2023.22 (PMC10600567; doi:10.1017/ehs.2023.22)
Supplement: Supplementary file 1 [file ehssup.zip › S2513843X23000221sup001.pdf]

Supplementary Material S2: Random Sampling Procedure and Schedule

The Random Sample (RS) section represents 15% of the manosphere discourse corpus, namely 1350 pages[1]. It is divided equally between the five manosphere branches. For each branch, the random sample is therefore composed of 270 pages. 90% of this sample was selected in 2021 (on all content up to 2021), 5% in 2022 (on content from 2021) and the remaining 5% in 2023 (on content from 2022), as detailed below in Figure S2.

Figure S2: Random Sample Architecture

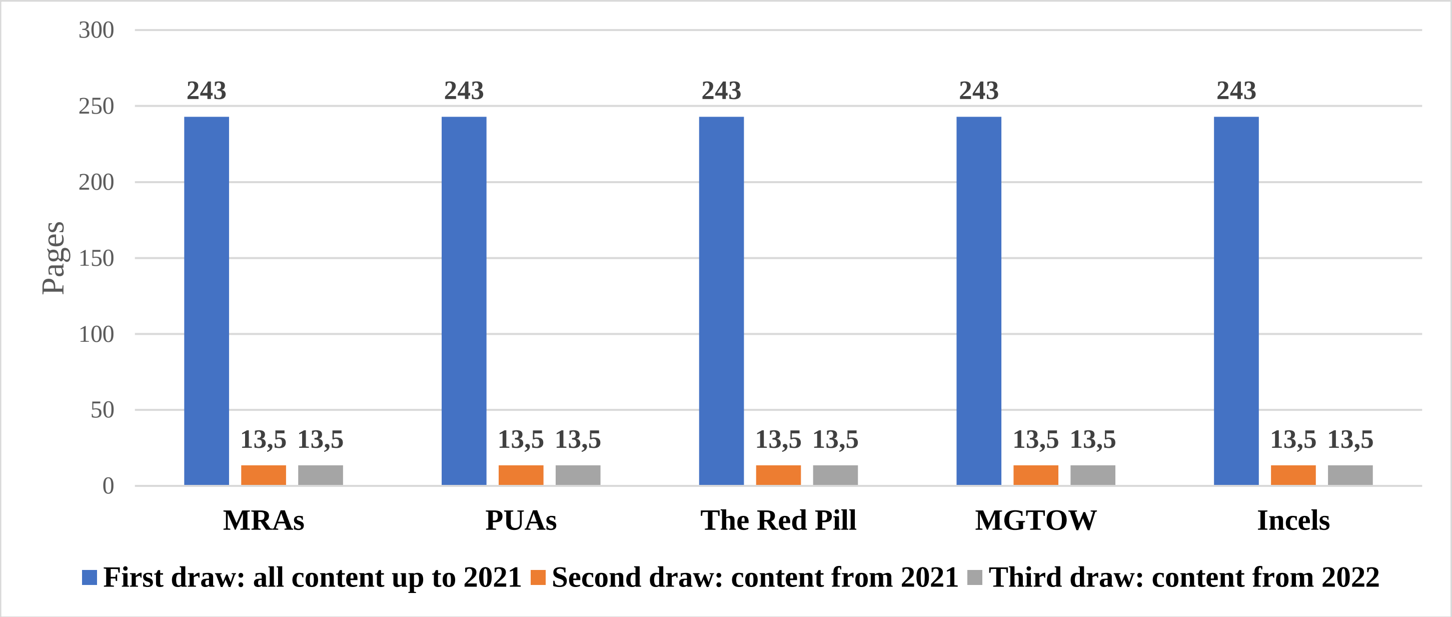

These are target numbers of pages. Threads and posts were therefore selected until the total number of pages exceeded the target. Then, the last post drawn was either included or rejected, based on the option that most approached the target number of pages. In case of a tie, the last post was not included. For actual number of pages of the randomly drawn material, see Supplementary Table S1.

Inclusion Criteria

This random sample is meant to represent the base, the everyday discussions of the manosphere. Therefore, the random sampling was carried out on websites and platforms where content is user-generated with no editing or selection for publication, i.e. forums and subreddits. From all the platforms included in the QC section (see Supplementary Table S1) all those which fit that criterion were selected. Content also needed to be easily accessible, navigable, and countable[2].

[1] Please note that all page numbers are +/- 5% since it is impossible to guarantee that documents conform to an exact length, especially if they are to be included in their entirety.
[2] Older websites that have been shut down are only very partially archived and almost impossible to navigate properly. Thus, the A Voice for Men, Roosh V, and The Attraction forums were excluded from the Random Sample section, as well as the banned r/incels subreddit.

Websites rarely come equipped with a "Random Page" feature; it thus needs to be engineered. There are two types of website architectures in the selected material: forums and subreddits, each requiring different methods.

Sampling Procedure: Forums

On forums, threads are contained in broader sections. Fortunately, the number of threads in each section is traditionally indicated. Thus, one only needs to add these figures to obtain the total number of threads. Each thread can therefore be considered to have an index number, for example in descending order. This is all that is required to select random threads, in this case with a simple spreadsheet. Threads were randomly drawn by their index numbers, then manually retrieved until the desired number of pages was reached. Each forum features some "utility" sections (such as the forum rules) which do not provide much information on people's ideas and beliefs. These utility sections were consequently removed from the threads pool before the random draw.

Sampling Procedure: Reddit

Apart from forums, most of the user-generated content in the manosphere comes from the website Reddit. Reddit's architecture makes random selection impossible to complete without more elaborate tools. In order to retrieve necessary data from the website, such as number of posts in a given subreddit, one needs to use an API (Application Programming Interface). The most popular Reddit API in social science research is called Pushshift[3]:

> "Pushshift is a social media data collection, analysis, and archiving platform that since 2015 has collected Reddit data and made it available to researchers. Pushshift's Reddit dataset is updated in real-time, and includes historical data back to Reddit's inception.[4]"

Using the Pushshift API, it was possible to run an algorithm selecting random Reddit posts on a given period, on a given subreddit. Reddit has a unit called "karma" which reflects a post's popularity. To avoid spams and junk submissions while still maintaining the largest pool of messages, only posts with karma superior to one were selected. Once selected via the algorithm, the threads were manually retrieved from the Reddit website until the desired number of pages was reached.

Threads and posts were always drawn, retrieved, and analyzed in their entirety. Indeed, those are discussions, where comments and posts reply to one another. We therefore avoided isolating or splicing such material, which might lead to flawed interpretations.

---

[3] Please note that as of writing (July 2023), Pushshift and other third-party APIs have been denied unlimited data access by Reddit; free and open use of those APIs might be permanently discontinued.

[4] Jason Baumgartner et al., "The Pushshift Reddit Dataset," *Proceedings of the Fourteenth International AAAI Conference on Web and Social Media (ICWSM 2020)*, (2020): 830–39, https://doi.org/10.1609/icwsm.v14i1.7347, 830.
