# Supplementary figures and images for: The use and misuse of evolutionary psychology in online manosphere communities: The case of female mating strategies
Source: Evol Hum Sci. 2023 Aug 30;5:e28. doi: 10.1017/ehs.2023.22 (PMC10600567; doi:10.1017/ehs.2023.22)

Figure S3: Manosphere Corpus Material Selection Process

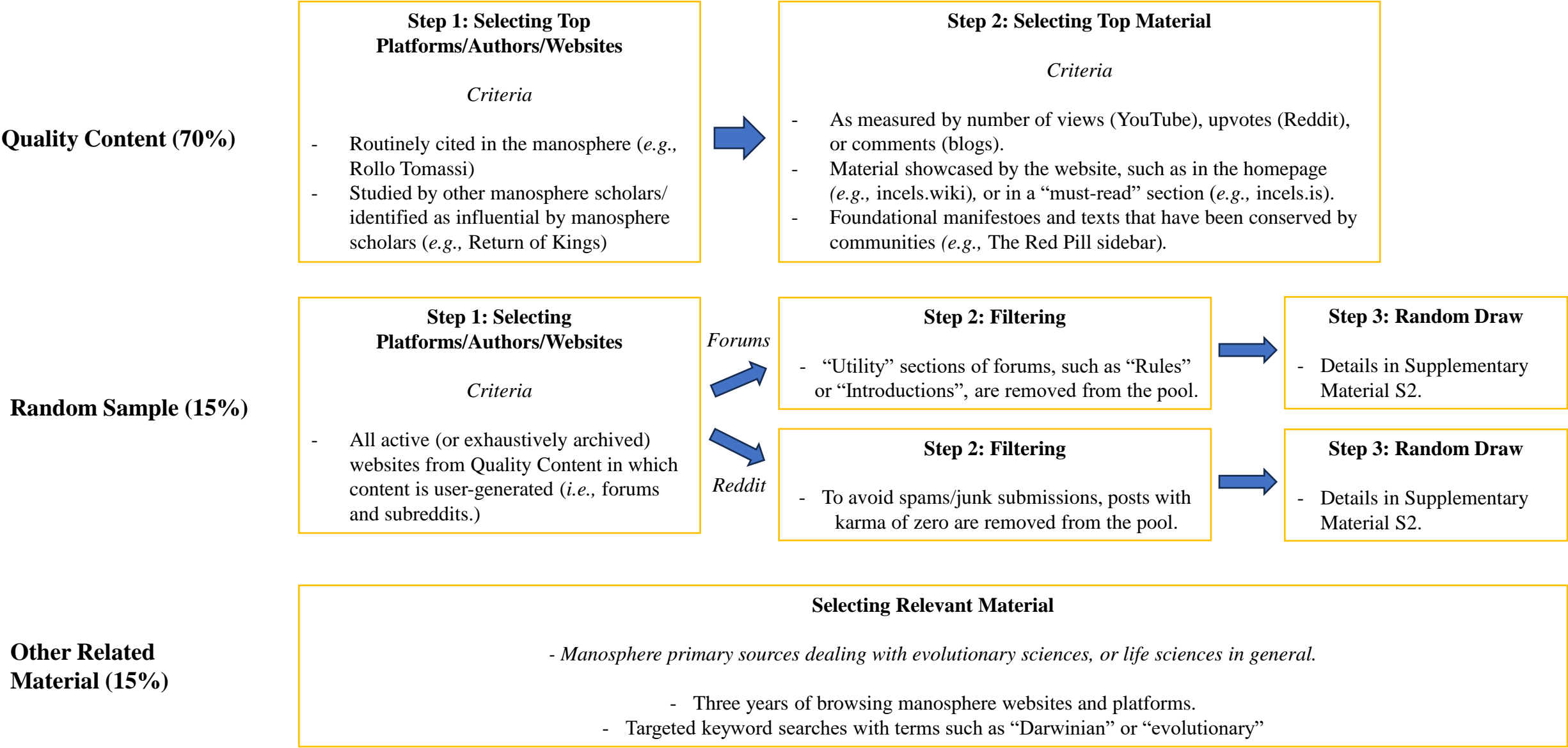

Supplement: Supplementary file 1 [file ehssup.zip › S2513843X23000221sup002.pdf]
